# Editorial for Special Issue “Cerebrovascular Diseases—From Pathogenesis to Treatment: Opportunities and Challenges”

**DOI:** 10.3390/cimb47120985

**Published:** 2025-11-26

**Authors:** Teresa Gasull, Francisco Purroy, Adrià Arboix

**Affiliations:** 1 Cellular and Molecular Neurobiology (CMN) Research Group at Germans Trias i Pujol Research Institute, 08916 Badalona, Catalonia, Spain; tgasull@igtp.cat; 2 Neurology Department, Stroke Unit, Hospital Universitari Arnau de Vilanova de Lleida, Universitat de Lleida, Biomedical Research Institute of Lleida, 25198 Lleida, Catalonia, Spain; fpurroy.lleida.ics@gencat.cat; 3 Cerebrovascular Division, Department of Neurology, Hospital Universitari del Sagrat Cor, Universitat de Barcelona, 08916 Barcelona, Catalonia, Spain

Stroke remains a leading cause of death and disability and has a complex pathophysiology [1,2]. Increasing evidence suggests that the brain is highly sensitive to even short-duration ischemia and that multiple mechanisms are involved in the tissue damage that results from cerebral ischemia. Ischemic stroke initiates a cascade of events, including ATP depletion, ionic dysregulation, increased release of glutamate, excess production of free radicals, edema, and inflammation; all of these events eventually contribute to cell death. In contrast, in intracerebral hemorrhage, the compression and destruction of brain tissue by a hematoma are the primary causes of brain injury, but inflammation, the coagulation response, and released hemoglobin toxicity play pivotal roles as well. Cell death after stroke has mainly been attributed in the past to necrosis or apoptosis, but recent reports show the involvement of other newly described forms of cell death [3,4,5,6,7,8].

This Special Issue, entitled “Cerebrovascular Diseases—From Pathogenesis to Treatment: Opportunities and Challenges”, aims to highlight some of the most novel pathophysiological and molecular aspects of acute stroke, emphasizing neurological biomarkers; cerebrovascular risk factors; the molecular and clinical implications of sleep-related breathing disorders for treating and recovering from acute stroke; therapeutic approaches targeting neuroinflammation, oxidative stress, and endothelial dysfunction in cerebral small vessel disease; as well as the glycoprotein IIIa PlA1/A2 polymorphism’s role in cerebrovascular disease risk, areas in which recent advances have been made. However, despite significant advancements, several controversies persist in the field of acute stroke mechanisms, pathophysiology, and treatment.

Following an excellent response from researchers around the world, the contributions listed below come from nine countries (China, Malaysia, Russia, the USA, Romania, the UK, Türkiye, Spain, and South Africa), reflecting the truly global and collaborative nature of contemporary cerebrovascular research.

Twelve manuscripts were submitted for consideration for the Special Issue, and all were subject to the rigorous *Current Issues in Molecular Biology* review process. In total, ten papers were ultimately accepted for publication and inclusion in this Special Issue (see list of contributions).

In this Special Issue, five original articles (contributions 1, 2, 4, 8, and 10) and five updated review papers (contributions 3, 5–7, and 9) were included (Table 1). In this Issue, we have collated a diverse set of research articles that highlight the importance of understanding the molecular signaling pathways involved in stroke.

Examining the latest evidence on this topic, a total of six articles analyzed cerebral infarctions (contributions 4 and 6–10), while ischemic and hemorrhagic strokes were analyzed in three studies (contributions 2, 3, and 5) and hemorrhagic stroke alone was analyzed in another study (contribution 1).

Emphasis was placed on the role of transmembrane 4 L six family member 1 (TM4SF1) in embryonic blood vessel development and hemorrhagic stroke pathophysiology (contribution 1) and glycoprotein IIIa PlA1/A2 polymorphism in the risk of acute cerebrovascular diseases (contribution 2). Also examined were the role of the dipeptidyl peptidase IV in post-COVID-19 cerebrovascular complications (contribution 3) and the analysis of the major blood lipid spectrum parameters in atherosclerosis and brain tumors as well as the assessment of correlations between these indicators and the Ki-67 proliferative activity index of the cells involved (contribution 4). The role of HSP47 in thrombotic disorders was evaluated (contribution 7). The molecular and clinical implications of sleep-related breathing disorders on treating and recovering from acute stroke (contribution 5) and the therapeutic approaches targeting neuroinflammation, oxidative stress, and endothelial dysfunction in cerebral small vessel disease (contribution 6) were also reviewed. Finally, a therapeutic approach using isoliquiritigenin (a type of chalcone that is widely found in medicinal plants of the Fabaceae family) in ischemic stroke was evaluated (contribution 8), and the relevance of prediabetes as a potential cerebrovascular risk factor was also evaluated (contributions 9 and 10).

The goal of this Monographic Issue is to provide a critical overview of the underlying factors involved in stroke-related brain injury, as well as the recent advances and controversies in this topic. Lin C.-I. et al., in their experimental study (contribution 1], demonstrated that the timeliness of reaching an optimal level of transmembrane 4 L six family member 1 (TM4SF1) expression to support TMED formation (TM4SF1-enriched microdomain) for endothelial cell and mesenchymal stem cell signaling and intercellular interactions is of critical importance during embryonic blood vessel development, preventing intraventricular and subarachnoid hemorrhage.

In a systematic search and meta-analysis, Coadă et al. (contribution 2) critically emphasized that the carrier status for the PIA2 polymorphism was significantly associated with a marginally elevated risk of ischemic stroke, though no significant association was found with hemorrhagic stroke.

Che Mohd Nassir et al., in their narrative review (contribution 3), showed that dipeptidyl peptidase IV is a main mediator in metabolic–inflammatory cascades, leading to the aggravation of post-COVID-19 fatigue and cerebrovascular complications. Its participation in glucose metabolism, immune regulation, and endothelial dysfunction, therefore, places it at the heart of the chronic cerebrovascular outcomes that accompany COVID-19 in survivors, such as neurovascular damage, stroke, and cognitive decline.

In their comparative study, Obukhova et al. (contribution 4) showed that there were only significant differences in the parameter values between atherosclerosis sufferers and the control group in terms of their ratios of neutral lipids to cholesterol. Of the short-chain fatty acids, butyric acid was of greatest interest due to the significant difference in its levels compared to the control group in the blood of patients with meningiomas and of those with gliomas. However, these parameters cannot serve as preoperative, non-invasive predictors of the Ki-67 mitotic index level, as no significant differences were found between them for low- and high-grade anaplasias of brain tumors.

Moreover, the scoping review by Uscamaita et al. (contribution 5) showed that the relationship between sleep-related breathing disorders and cardiovascular conditions is significant, encompassing stroke (ischemic and hemorrhagic), hypertension, myocardial infarction, post-myocardial infarction heart failure, and atherosclerosis. Promptly recognizing sleep apnea in acute stroke patients could be important for guiding therapy and forecasting potential complications and long-term outcomes.

Yılmaz and Bayraktutan (contribution 6), using an updated review, analyzed therapeutic approaches targeting neuroinflammation, oxidative stress, and endothelial dysfunction in cerebral small vessel disease and concluded that there is a need to deepen the understanding of the molecular pathologies underlying cerebral small vessel disease and explore potential pharmacological interventions through well-designed preclinical studies targeting neuroinflammation, oxidative stress, and endothelial dysfunction.

In their systematic literature review, Teodoru et al. (contribution 7) remarked that HSP47, a key collagen-specific molecular chaperone, plays a vital role in collagen maturation, folding, and stabilization in humans. Its activity is crucial for maintaining the stability and assembly of collagen types essential to connective tissue health. While primarily functioning within the endoplasmic reticulum, HSP47’s influence extends beyond cellular protein synthesis, impacting adiposity regulation and cardiovascular health.

Lastly, Yuan et al. (contribution 8) emphasized that isoliquiritigenin may exert an anti-ischemic stroke effect through regulating APP (amyloid precursor protein), ESR1 (estrogen receptor alpha), MAO-A (monoamine oxidase type-A), and PTGS2 (prostaglandin-endoperoxidase synthase).

The links between prediabetes and acute stroke were analyzed by Naicker and Khathi (contributions 9 and 10).

Overall, the research presented in this Special Issue underscores the complex nature of stroke and the importance and necessity of a multidisciplinary research approach to advance the understanding of stroke pathophysiology [9,10,11,12,13,14,15,16,17,18].

In summary, in this Special Issue, the authors highlight a number of research directions and topics with the aim of studying the molecular and cellular mechanisms of acute stroke. Improving our understanding of the underlying molecular mechanisms of cerebrovascular diseases is imperative for further integrating innovative drug, cell, and gene therapy approaches into carefully controlled clinical trials, and will promote significant advances in a field with many unmet needs.

We hope that the diverse perspectives and methodological innovations showcased in this Special Issue will be both stimulating and valuable to our readers, inspiring collaborative efforts to advance the field of molecular biology in cerebrovascular research.

Open lines of research include the pathophysiological and molecular aspects of acute stroke in octogenarians; differences between men and women; and comparing cardioembolic versus atherothrombotic strokes or lacunar versus non-lacunar infarctions—all of which are important and challenging subsets of acute cerebrovascular diseases that should be evaluated in further investigations.

We would like to extend our sincere thanks to all the authors, peer reviewers, and editors who contributed to this Special Issue.

## Figures and Tables

**Table 1:** Analysis of the published contributions included in the present Special Issue “Cerebrovascular Diseases—From Pathogenesis to Treatment: Opportunities and Challenges”.

N#*	Authors	I/H	Stroke Relevance	Topic	Characteristics of the Article	Conclusions
1	Lin C-I et al.	H	Hemorrhagic stroke pathophysiology (intraventricular and subarachnoid hemorrhage)	Role of the transmembrane-4L-six family (TM4SF1) in embryonic blood vessel development.	Experimental study	This study reveals that the timeliness of reaching an optimal TM4SF1 expression level to support TMED formation for endothelial cell and mesenchymal stem cell signaling and intercellular interactions is of critical importance during embryonic blood vessel development.
2	Coadä et al.	I/H	Cerebrovascular risk factors	Role of the glycoprotein IIIa PlA1/A2 polymorphism in the risk of cerebrovascular diseases.	Systematic search and meta-analysis	The carrier status for the PIA2 polymorphism was significantly associated with a marginally elevated risk of ischemic stroke. No significant association could be found with hemorrhagic stroke.
3	Che Mohd Nassir et al.	I/H	Chronic cerebrovascular dysfunctions that follow COVID-19	Dipeptidyl peptidase IV’s role in post-COVID-19 cerebrovascular complications.	Narrative review	Dipeptidyl peptidase IV is a main mediator in metabolic–inflammatory cascades leading to the aggravation of post-COVID-19 fatigue and cerebrovascular complications. Its participation in glucose metabolism, immune regulation, and endothelial dysfunction, therefore, places it at the heart of the chronic cerebrovascular outcomes that accompany COVID-19 survivors, such as neurovascular damage, stroke, and cognitive decline.
4	Obukhova et al.	I	Cerebrovascular risk factors (blood lipid spectrum)	Analysis of the major parameters of the blood lipid spectrum in atherosclerosis and brain tumors, as well as assessing any correlations of these indicators with the Ki-67 proliferative activity index of the cells involved.	Comparative clinical and biological study	Significant differences were only found in the parameter values between the atherosclerosis sufferers and the control group for their ratios of neutral lipids to cholesterol. Of the short-chain fatty acids, butyric acid is of the greatest interest due to the significant differences in its levels in the blood of patients with meningiomas and gliomas from the control group. However, these parameters cannot serve as a preoperative non-invasive predictor of the Ki-67 mitotic index level, as no significant differences between them were found for low- and high-grade anaplasia of brain tumors.
5	Uscamaita et al.	I/H	Sleep-related breathing disorders as a cerebrovascular risk factor	Molecular and clinical implications of sleep-related breathing disorders on treating and recovering from acute stroke.	Scoping review	The relationship between sleep-related breathing disorders and cardiovascular conditions is significant, encompassing stroke (ischemic and hemorrhagic), hypertension, myocardial infarction, post-myocardial infarction heart failure, and atherosclerosis. The prompt recognition of sleep apnea in acute stroke patients could be important for possible therapy and forecasting potential complications and long-term outcomes.
6	Yılmaz and Bayraktutan	I	Therapeutic approach in cerebral small vessel disease	Therapeutic approaches targeting neuroinflammation, oxidative stress, and endothelial dysfunction in cerebral small vessel disease.	Updated review	A list of drugs were analyzed to prevent neuroinflammation and oxidative damage and effectively maintain endothelial function and blood–brain barrier integrity, including anti-anginal drugs, acetylcholine esterase inhibitors, β-hydroxy β-methylglutaryl-CoA reductase inhibitors, lithium drugs, phosphodiesterase inhibitors, oral antihyperglycemic drugs, and tetracycline antibiotics. However, it is necessary to improve the understanding of the molecular pathologies underlying cerebral small vessel disease and explore potential pharmacological interventions through well-designed preclinical studies targeting neuroinflammation, oxidative stress, and endothelial dysfunction.
7	Teodoru M. et al.	I	Role of HSP47 in thrombotic disorders	Relevant in vitro and in vivo studies examining platelet aggregation, collagen–HSP47 interactions, and thrombus formation.	Systematic literature review	Reveals the emerging significance of heat shock protein 47 (HSP47) in thrombotic disorders, particularly deep vein thrombosis (DVT) and atherosclerosis. HSP47 facilitates platelet–collagen interactions critical for thrombus formation and vascular stability in vulnerable plaques.
8	Yuan et al.	I	Therapeutic approach of using isoliquiritigenin in ischemic stroke	An integrated network pharmacology, molecular docking, molecular dynamics simulation, and experimental validation study was performed to investigate the potential mechanism of isoliquiritigenin in treating ischemic stroke.	Experimental study	The report emphasizes that isoliquiritigenin may exert an anti-ischemic stroke effect by regulating APP, ESR1, MAO-A, and PTGS2, determined according to the combined results of network pharmacology, molecular docking, molecular dynamics simulation, and experimental validation.
9	Naicker and Khathi	I	Cerebrovascular risk factors (prediabetes and stroke)	Links between prediabetes and stroke.	Systematic literature review	Individuals with prediabetes may show alterations in inflammatory (IL-6), coagulation (D-dimer), and neurovascular (S100B, GFAP, NSE) markers, though some of these findings are inconsistent, reflecting early pathophysiological changes that may increase stroke risk.
10	Naicker and Khathi	I	Cerebrovascular risk factors (prediabetes and stroke)	Relevance of prediabetes as a cerebrovascular risk factor.	Comparative clinical and biological study	Inflammatory, coagulation, and neurovascular biomarkers, particularly S100B, may indicate early stroke risk in prediabetes.

I: ischemic stroke; H: hemorrhagic stroke; N#*: number of contributions.
